# Pro-inflammatory interleukin-18 increases Alzheimer’s disease-associated amyloid-β production in human neuron-like cells

**DOI:** 10.1186/1742-2094-9-199

**Published:** 2012-08-16

**Authors:** Elina M Sutinen, Tuula Pirttilä, George Anderson, Antero Salminen, Johanna O Ojala

**Affiliations:** 1 University of Eastern Finland, Institute of Clinical Medicine/ Neurology, Canthia, P.O.B. 1627, FI-70211, Kuopio, Finland; 2 University of Eastern Finland, Clinical Research Centre/ Brain Research Unit, Mediteknia, P.O.B. 1627, FI-70211, Kuopio, Finland; 3 CRC, Rm30, 57 Laurel Street, Glasgow, Scotland, UK; 4 Kuopio University Hospital, Neurology, P.O.B. 1777, FI-70211, Kuopio, Finland

**Keywords:** Interleukin-18, Inflammation, Amyloid-beta, Alzheimer’s disease, BACE, Presenilin

## Abstract

**Background:**

Alzheimer’s disease (AD) involves increased accumulation of amyloid-β (Aβ) plaques and neurofibrillary tangles as well as neuronal loss in various regions of the neocortex. Neuroinflammation is also present, but its role in AD is not fully understood. We previously showed increased levels of pro-inflammatory cytokine interleukin-18 (IL-18) in different regions of AD brains, where it co-localized with Aβ-plaques, as well as the ability of IL-18 to increase expression of glycogen synthase kinase-3β (GSK-3β) and cyclin dependent kinase 5, involved in hyperphosphorylation of tau-protein. Elevated IL-18 has been detected in several risk conditions for AD, including obesity, type-II diabetes, and cardiovascular diseases as well as in stress.

**Methods:**

We differentiated SH-SY5Y neuroblastoma cells as neuron-like and exposed them to IL-18 for various times. We examined the protein levels of amyloid-β precursor protein (APP) and its processing products, its cleaving enzymes, involved in amyloidogenic processing of APP, and markers of apoptosis.

**Results:**

IL-18 increased protein levels of the β-site APP-cleaving enzyme BACE-1, the N-terminal fragment of presenilin-1 and slightly presenilin enhancer 2, both of which are members of the γ-secretase complex, as well as Fe65, which is a binding protein of the C-terminus of APP and one regulator for GSK-3β. IL-18 also increased APP expression and phosphorylation, which preceded increased BACE-1 levels. Further, IL-18 altered APP processing, increasing Aβ40 production in particular, which was inhibited by IL-18 binding protein. Increased levels of soluble APPβ were detected in culture medium after the IL-18 exposure. IL-18 also increased anti-apoptotic bcl-xL levels, which likely counteracted the minor increase of the pro-apoptotic caspase-3. Lactate dehydrogenase activity in culture medium was unaffected.

**Conclusions:**

The IL-18 induction of BACE-1, APP processing, and Aβ is likely to be linked to stress-associated adaptations in neurons during the course of normal functioning and development. However, in the course of wider changes in the aging brain, and particularly in AD, the effects of heightened or prolonged levels of IL-18 may contribute to the process of AD, including via increased Aβ.

## Background

Alzheimer’s disease (AD) is a neurodegenerative disorder, which is characterized particularly by accumulation of amyloid-β (Aβ) peptide in the brain parenchyma and in the walls of leptomeningeal and parenchymal vessels [1]. Neurofibrillary changes, neuronal loss, synaptic dysfunction, evidence of oxidative and excitotoxic damage as well as low grade inflammation are also present in the AD brain [2-4]. Increased Αβ production from intracellular amyloid precursor protein (APP) metabolism, are believed to initiate the pathogenic processes in AD. Inflammation also seems to have a role in the ethiopathogenesis of AD [4,5], although it is not clear whether inflammation is a cause, contributor, or secondary phenomenon in the disorder [6]. There is also no clear causal connection between the up-regulation of pro-inflammatory cytokines and Aβ expression.

Over-expression of pro-inflammatory interleukins 1 and 6 (IL-1, IL-6) has been detected in the AD brain [7-10]. Pyrogenic IL-1β can induce over-expression of APP [11] contributing to the generation of neuritic Aβ-plaques. We have found increased levels of another IL-1 family member, pro-inflammatory IL-18 (interferon-γ-inducing factor, IL-1γ), in AD brain [12]. Non-pyrogenic IL-18 has structural similarities with the IL-1 family of proteins and it can increase production of toxic inflammatory molecules such as IFN-γ [13] and IL-1β [14]. IFN-γ, via caspase-1 induction, cleaves the pro-forms of IL-1β and IL-18 to their active mature forms [15] and also increases levels of IL-18 [16], indicating that up-regulated IL-18 expression can lead to a harmful vicious cycle of inflammation, possibly also in the brain. IL-18 is produced primarily by activated microglia, but also by astrocytes and ependymal cells in the central nervous system (CNS) [17,18]. IL-18 is also detectable in neurons [18,19].

The IL-18 gene is located in the region 11q22.2-22.3 close to the dopamine receptor D2 locus. This region is near the tip of the long arm of chromosome 11, which has been reported as a suggestive linkage area for AD in AD families [20,21]. Further, the IL-18 promoter contains multiple transcription initiation sites [16], and its polymorphism has been shown to be a risk of late onset AD [22,23]. However, only single nucleotide polymorphisms (SNPs) at positions −137 and −607 have been confirmed to have an impact on IL-18 gene activity [24,25]. Individuals with two copies of the IL-18 -607 C or −137 G allele have been found to have increased odds of developing AD and these associations were influenced by the presence of the ApoE ϵ4 alleles, increasing the risk to develop AD more than five- and four-fold, respectively [23]. These alleles as well as −607 C/-137 G haplotype are associated with higher expression of the IL-18 gene and sustained higher levels of the cytokine [23]. However, Segat *et al*. (2010) could not find association between IL-18 gene promoter polymorphism (−137 G/C and −607 C/A) and onset of AD [26].

Although no significant increase of circulating cytokines has been detected in AD patients [27], the levels of IL-18 mRNA in monocyte-macrophages of the peripheral blood has been shown to be high in AD-mild patients, slightly lower in AD-moderate patients, but not significantly different in AD-severe patients compared to non-demented age-matched subjects [28]. On the other hand, a significant increase in production of IL-18 has been obtained from lipopolysaccharide (LPS)-stimulated blood mononuclear cells of AD patients, and that was particular in AD patients carrying the −607 C/C genotype of IL-18 promoter. In addition, a significant correlation between IL-18 production and cognitive decline was apparent in AD patients [27]. Therefore, the increase in the potency of these cells to respond to stimulation by LPS in AD patients may reflect the relative increase of inflammatory response. This may also occur in the brain in response to, for example, Aβ, since Aβ42 has been shown to prime at least dendritic cells to enhanced release of IL-1β, IL-6, and IL-18 [29].

Binding of IL-18 to its receptor in the CNS leads to activation of the transcription factor NF-κB via a complex intracellular cascade [30]. Via NF-κB activation, IL-18 can also activate both Fas and Fas-L promoter activities [31], which suggests the possibility that IL-18 may be one of the apoptosis-inducing factors contributing to neurodegeneration during AD progression. IL-18 can also modulate neuronal excitability [32] and IL-18, like IL-1β and TNF-α, inhibits long-term potentiation (LTP), a form of neuronal plasticity widely believed to underlie learning and memory, both in the early p38 mitogen activated protein kinase-dependent (MAPK p38) phase and the later protein synthesis-dependent phase [33].

The importance of IL-18 in APP synthesis and processing is unknown. Interestingly, increased levels of IL-18 have been found in the blood of patients with ischemic heart disease [34], type-2 diabetes, and obesity [35], which are all known risk factors for AD [36,37]. The links between these disorders and AD is not fully understood. IL-18 is also associated with stress [38] and regulates anxiety and fear memory in mice [39]. IL-18 levels are generally increased in Cushing’s disease patients, where a significant positive correlation between IL-18 and cortisol is evident [40]. Stress hormones can also increase IL-18 synthesis at least in the layers of zona reticularis and zona fasciculate in the adrenal glands and peripheral mononuclear cells [40]. IL-18 secretion can be inhibited, at least in dendritic cells, with the calcium-channel blocker nifedipine [41].

**Figure 1:**
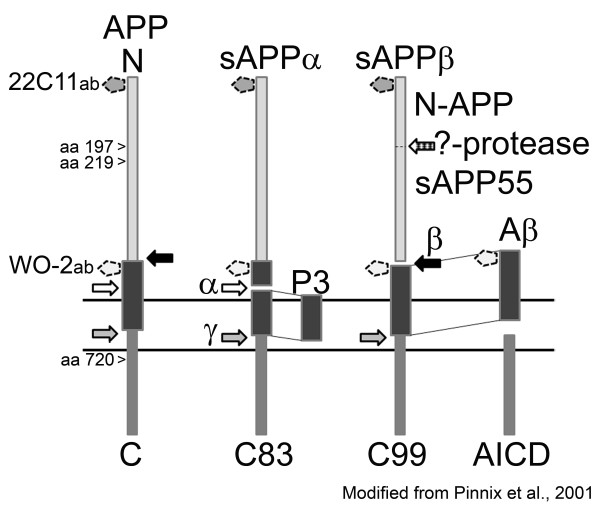
**Processing of APP by secretases.** APP (85 kD) can be cleaved by α-secretase (α). This generates soluble APPα (sAPPα; 76 kD) and C-terminal fragment C83 (CTFα; ~9 kD). In the amyloidogenic pathway, APP is cleaved by β-secretase (β) to generate of sAPPβ (74 kD) and the C-terminal fragment C99 (CTFβ; approximately 11 kD), which in turn is cleaved by γ-secretase (γ) to generate amyloid-β (Aβ; approximately 4.5 kD) and amyloid intracellular domain AICD (CTFγ; approximately 6 kD). AICD is also produced when C83 is cleaved by γ-secretase [46]. This cleavage generates also P3 (approximately 2.6 kD). The N-terminal fragment of sAPPβ can be cleaved by an as yet unconfirmed protease as N-APP (approximately 30 kD) and sAPP55 (approximately 45 kD) [51]. Arrowheads indicate caspase-3 cleavage site of APP amino acids (aa) [52]. WO-2 and 22C11 are used in this study as antibodies (ab) to detect the shown fragments. The dotted line pentagons indicate the binding sites of the antibodies.

APP is an integral membrane glycoprotein with receptor-like structure [42,43], but its physiological functions, and those of its cleavage products, are still to be fully clarified. Proliferating cells express APP, and it is detectable at the earliest stages of embryonic development [44,45]. As described in Figure 1, the cleavage products of APP can be soluble N-terminal fragments APPα (sAPPα), APPβ (sAPPβ), as well as Aβ and different sized C-terminal fragments (CTFs), depending on whether APP is cleaved by α-, β-, or γ-secretases, and the cleavage order [46]. In the amyloidogenic Aβ-producing pathway, APP is first cleaved by transmembrane protease β-site amyloid precursor protein-cleaving enzyme (BACE, β-secretase) [47], localized within Golgi, endosomes, and plasma membrane [48] and then by intramembrane γ-secretase. γ-secretase is a multiprotein complex containing a catalytic site harboring endoproteolytically cleaved N- and C-terminal fragments of presenilin-1 (PS-1), nicastrin, anterior pharynx defective 1 (Aph1), and presenilin enhancer 2 (Pen-2). This complex forms in the Golgi/trans-Golgi network [49]. In the non-amyloidogenic pathway, APP is cleaved by α-secretase. A Disintegrin and Metalloproteinase domain-containing proteins 9, 10, and 17 (ADAM9, ADAM10, ADAM17/TACE; TNF-α convertase) can function as an α-secretase [50].

The amyloidogenic processing of APP seems to be at least partially regulated by its Thr668 phosphorylation (numbering for APP695 isoform) [53,54], including by the cyclin-dependent kinase 5 (Cdk5)/p35 complex [55]. Previously we showed that IL-18 increases not only protein levels of Cdk5/p35 but also glycogen synthase kinase-3β (GSK-3β) [19]. GSK-3β seems to be regulated at least partially by the Fe65/AICD complex [56]. GSK-3β phosphorylates and regulates the levels and function of PS-1 [57,58].

In this study, we examined the relationship of IL-18 on APP processing and Aβ formation *in vitro* in neuron-like differentiated SH-SY5Y-neuroblastoma cells. We found that IL-18 increased production of APP and its phosphorylation at Thr668. Further, IL-18 increased the amyloidogenic processing of APP by increasing expression of BACE-1 (70 kD) and levels of the 34 kD N-terminal fragment of PS-1 (NTF), contributing to a functional γ-secretase. Expression of ADAM10 was very low in these cells, thus definite conclusions as to the impact of IL-18 cannot be made. Increased Aβ production after IL-18 administration was detectable in cell lysate, and this was prevented by the simultaneous application of IL-18 binding protein (IL-18 bp). Increased Aβ production was also detectable in normal human astrocytes (NHA) after the addition of IL-18. IL-18 increased cellular level of bcl-xL but had only a minor impact on cellular caspase-3 and did not affect lactate dehydrogenase (LDH) activity in culture medium. Some comparison studies were done with IL-1β, and the results were somewhat in line with the IL-18 results, although the timeframe of changes seemed to be different.

## Materials and methods

### Cell culture

The work was done with commercially available cells and products only. SH-SY5Y neuroblastoma (DSMZ; Braunschweig, Germany) were maintained in 50% Dulbecco’s medium (DMEM; BioWhittaker/Lonza) containing 4.5 g/L glucose and 50% Opti-MEM® Reduced Serum Medium with GlutaMAX™ (Invitrogen). Mixture was supplemented with 5% heat-inactivated fetal bovine serum (iFBS; HyClone/Pierce; Logan, UT, USA), 1 mM L-glutamine (Lonza), 100 U/mL penicillin and 10 μg/mL streptomycin (Lonza). The cells were plated as 10^5^ cells/well into the 12-well plates (Nunc™; Roskilde, Denmark) in DMEM containing the supplements. The cells were differentiated for 3 days with 10 μM all-trans retinoic acid (ATRA; Sigma-Aldrich; St Louis, MO, USA) treatment, which was followed by 4 days of 50 ng/mL human recombinant brain-derived neurotrophic factor (BDNF; Alomone Labs, Jerusalem, Israel) treatment in DMEM with supplements but without iFBS. Normal human astrocytes (NHA; Lonza) were plated as 6 × 10^4^ cells/well in 12-well plates and they were grown in DMEM containing 4.5 g/L glucose, and supplemented with 10% iFBS, 2 mM L-glutamine, antibiotics, and 1× AGM^TM^ SingleQuots® (Lonza). The NHA experiments were initiated 2 days after plating.

### Treatments

Recombinant human IL-18 (IL-18; MBL, Naka-ku Nagoya, Japan) was added to the culture medium as described [19]. IL-18 binding protein (IL-18 bp; Recombinant Human IL-18BPa/Fc Chimera; R&D Systems) was added at 0.5 μg/mL to the pre-made IL-18 (100 ng/mL) medium, and the mixture was added immediately to the cultures. BDNF was present in SH-SY5Y cultures during the IL-18 treatments. IL-1β (Biosource/Invitrogen) was used at 10, 20, 50, 100, 150 ng/mL in some comparison SH-SY5Y experiments. NHA cells were treated with 100 ng/mL IL-18, IL-1β (Biosource/Invitrogen), or Interferon-γ (Biosource/Invitrogen) for 6 h or exposed to 1 mM H_2_O_2_ (J.T.Baker; Deventer, Holland) in DMEM for 10 min, after which fresh culture medium was added for 6 h. Treatments were done without iFBS and AGM^TM^ SingleQuots® supplementation. Amyloid β-Protein (1–42) was purchased from Bachem (Bachem AG, Bubendorf, Switzerland). Some of the SH-SY5Y experiments were photographed just before harvesting by using Nikon Ecclipse TE300 inverted tissue culture microscope (objective Plan Fluor 20×/0.45) and Nikon Coolpix 995 camera. After the incubations, culture media were collected and the cells were lysed with M-PER lysis buffer (Pierce/Thermo Fisher Scientific Inc., Rockford, IL, USA) containing Protease Inhibitor Mix (GE Healthcare).

### Protein, LDH, and ELISA measurements

Protein levels were estimated with the DC protein assay kit (Bio-Rad Laboratories, Hercules, CA, USA) according to manufacturer’s protocol. LDH activity was measured from SH-SY5Y culture medium with CytoTox 96® Nonradioactive Cytotoxicity Assay (Promega). Aβ forms were examined from cell lysate and culture medium by using Amyloid Beta x-40 and Amyloid Beta x-42 Chemiluminescent ELISA kits (Covance/Nordic Biosite) according to manufacturer’s instructions.

### Immunoblotting

Equal protein amounts (20 μg or 35 μg) from lysate and equal medium volumes (65 μL) were separated in 12.5% gels in sodium dodecyl sulfate polyacrylamide gel electrophoresis and the proteins were transferred to a Hybond P membrane (GE Healthcare). Full-Range and Low-Range Rainbow Molecular Weight Markers (GE Healthcare) as well as Novex® Sharp Prestained Protein Standard (Life Technologies™) were used for the detection of the correct molecular weight targets.

The following antibodies were purchased from Santa Cruz Biotechnology, Inc. (Santa Cruz, CA, USA): BACE-1 (61-3E7; sc-33711), PS-1 (H-70; sc-7860), Pen-2 (FL-101; sc-32946), Aph-1 (H-50; sc-98469), nicastrin (N-19; sc-14369), ADAM10 (S-10; sc-16523), Fe65 (H-260; sc-33155), p-β-Amyloid (Thr 668) (sc-101632), caspase-3 p11 (K-19; sc-1224), and bcl-xL (S-18; sc-634). Anti-Aβ[4-10]-Antibody (WO-2) was purchased from the Genetics Company, Inc. (Schlieren, Switzerland) and anti-Alzheimer precursor protein A4 antibody (22C11) from Chemicon/Millipore. Anti-Actin (H-196; sc-7210; Santa Cruz Biotechnology, Inc.) and anti-α-Tubulin (clone B-5-1-2, mouse Ascites fluid; Sigma-Aldrich) were used as loading controls.

The membranes were blocked with 3% non-fat milk (Valio; Finland) in phosphate buffered saline (PBS; pH 7.5)/0.05% Tween-20 (Sigma-Aldrich) for 1 h, and the antibodies purchased from Santa Cruz Biotechnology, Inc. were used as 1:300 or 1:400 dilutions and Tubulin as 1:8000 as described [19]. When 22C11 and WO-2 antibodies were used, the membranes were blocked with 5% non-fat milk and 0.25% bovine serum albumin, fraction V (Roche Diagnostics; Mannheim, Germany) in Tris-buffered saline (pH 7.6; TBS)/0.05% Tween-20, at room temperature for 30 min, after which the antibody (0.75 μg/mL) was added. Incubations were done overnight at +4 °C. After all antibody incubations, the membranes were washed as described [19].

For detection, the membranes were incubated with ECL™ donkey anti-rabbit IgG F(ab’)_2_ peroxidase-linked detection antibody (GE Healthcare), ECL™ anti-mouse IgG peroxidase-linked species-specific whole antibody (from sheep) (GE Healthcare) or bovine anti-goat IgG-HRP, mouse/human adsorbed (sc-2384; Santa Cruz Biotech.) detection antibodies at room temperature for 1 to 1.5 h. Immobilon Western Chemiluminescent HRP Substrate (Millipore; Billerica, MA, USA) was added to the washed membrane and Fujifilm Super RX (Fuji Photo Film Co., Ltd) was used for the documentation. The detected bands were quantitied with a MCID M5-image analysis system (Imaging Research Inc.; Ontario, Canada).

The membranes were stripped after the detection by washing four times with TBS/0.1% Tween-20, incubating in stripping buffer (62.7 mM Tris, 2% SDS and 0.7% β-mercaptoethanol; pH 6.8) slightly shaking at +50 °C, followed by washing six times with TBS/0.1% Tween-20. The membranes were used three to five times.

### Statistical analysis

The numerical results were analyzed with Mann–Whitney *U*-Test (M-W) and Student’s *t*-Test (*t*-Test). The experiments were independently repeated as indicated and the number (*n*) of controls and treatments were equal.

## Results

### IL-18 treatment increased protein levels of BACE-1, NTF of PS-1, and Fe65

**Figure 2:**
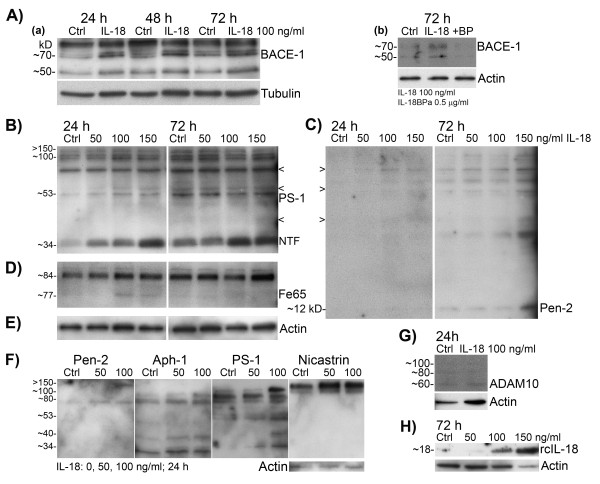
**Levels of APP cleaving enzymes in neuron-like differentiated SH-SY5Y neuroblastoma cells.** The cells were treated with IL-18 (50, 100, or 150 ng/mL) for described times. (**A**) BACE-1 (70 kD) increased after 100 ng/mL IL-18 treatment compared to the untreated control (a), (b); the impact of IL-18 was suppressed with IL-18 bp (IL-18 100 ng/mL + IL-18BPa 0.5 μg/mL) (b). (**B**) IL-18, in a concentration-dependent manner, increased protein level of NTF of PS-1 (34 kD) compared to the untreated control. Protein synthesis of PS-1 (53 kD) increased slightly only at 24 h in IL-18 treated cells. The bands above are likely partially formed γ-secretase complexes including NTF of PS-1. (**C**) IL-18 treatment increased expression of Pen-2 at 72 h. Arrowheads indicate bands in PS-1 and Pen-2 films co-localized when the Pen-2 and PS-1 films were superimposed. (**D**) Fe65 (77 kD) increased in 24-h treated cells. (**E**) Loading control actin for the results B to D, which were obtained from the same membrane. (**F**) Aph-1 (29 kD) and nicastrin increased in 24-h IL-18 treated cells. The probings are from the same membrane and arranged according to the superimposing. (**G**) Expression of ADAM10 was generally very low (60 kD active form, 80 kD partially processed, 100 kD precursor). (**H**) The presence of recombinant IL-18 in 72-h IL-18 treated SH-SY5Y lysate. Actin and Tubulin were used as loading references. Ctrl, untreated control; NTF, N-terminal fragment of PS-1; RcIL-18, recombinant IL-18.

First we examined the presence of IL-18 receptor (IL-18R) and IL-18 bp in differentiated SH-SY5Y neuroblastoma cells. Our cells expressed IL-18R and its protein levels were unaltered by IL-18 treatments (data not shown). IL-18 bp was not detectable at the protein level (data not shown). We also detected added correct size IL-18 in the cell lysate even in 72-h treated cells indicating its stability (Figure 2H). Our findings are in line with Sallmon *et al*. (2010), who have previously shown that SH-SY5Y neuroblastoma cells express IL-18R and IL-18 bp, and when differentiated with ATRA, also IL-18, whereas the level of IL-18 bp reduces after ATRA treatment [59].

We examined how IL-18 affects protein levels of particularly BACE-1 and PS-1 in neuron-like differentiated SH-SY5Y neuroblastoma. IL-18 (100 ng/mL) increased the protein levels of BACE-1 (70 kD) at 24 h (131%, *P* <0.000) and 72 h (68%, *P* <0.000) time points (Figure 2A(a),(b); Table 1), and the impact of IL-18 was suppressed with simultaneous treatment with 0.5 μg/mL IL-18 bp (Figure 2A(b), last lane). The effect of IL-18 on BACE-1 was also concentration (50–150 ng/mL) dependent particularly in 24-h treated cells (data not shown). Protein levels of NTF of PS-1 (34 kD) were also increased by IL-18 in a concentration dependent manner (50–150 ng/mL) (Figure 2B). The increase in NTF was significant at 6 h in IL-18 treated cells (192%, *P* 0.017) (Table 1). The 53 kD precursor of PS-1 was significantly increased later, at 48 h, following IL-18 addition (26%, *P* 0.068 (M-W), *P* 0.050 (*t*-Test)) (Table 1) with the increase at 24 h approaching significance (67%, *P* 0.058 (*t*-Test)) (Table 1).

**Table 1:** Mean percentage alteration of protein levels of IL-18 treated targets compared to untreated ones, analyzed from western blots

		**6 h**	**24 h**	**48 h**	**72 h**
APP, approximately 100 kD	Mean % ± SEM	**74.9** ±17.2	**19.3** ±9.9	**56.5** ±12.4	**−3.0** ±10.1
	*n*	20	31	30	31
	*t*-Test, *P*	***<0.000***^a^	*0.062*	***<0.000***^a^	*0.769*
	M-W, *P*	***0.001***^a^	*0.809*	***<0.000***^a^	*0.243*
BACE-1, approximately 70 kD	Mean % ± SEM	**35.1** ±29.7	**131.1** ±35.4	**39.1** ±25.1	**67.8** ±21.5
	*N*	11	20	18	19
	*t*-Test, *P*	*0.107*	***0.002***^b^	*0.310*	***0.006***^b^
	M-W, *P*	*0.700*	***<0.000***^a^	*0.543*	***<0.000***^a^
PS-1, approximately 34 kD	Mean % ± SEM	**191.9** ±80.7	**22.6** ±28.4	**26.5** ±25.9	**5.4** ±16.0
	*n*	7	17	16	18
	*t*-Test, *P*	*0.055*	*0.322*	*0.323*	*0.741*
	M-W, *P*	***0.017***^c^	*0.734*	*0.519*	*1.000*
PS-1, approximately 53 kD	Mean % ± SEM	**121.9** ±68.7	**67.0** ±32.9	**26.0** ±12.3	**−18.9** ±10.4
	*n*	9	18	18	18
	*t*-Test, *P*	*0.114*	*0.058*	***0.050***c	*0.087*
	M-W, *P*	*0.203*	*0.543*	*0.068*	*0.543*
PS-1, approximately 100 kD	Mean % ± SEM	**79.7** ±55.9	**51.4** ±19.2	**27.8** ±16.4	**8.1** ±12.1
	*n*	10	19	19	20
	*t*-Test, *P*	*0.106*	*0.138*	*0.767*	*0.247*
	M-W, *P*	*0.192*	***0.016***^c^	*0.106*	*0.377*
bcl-xL, approximately 30 kD	Mean % ± SEM	**20.7** ±36.6	**−6.3** ±11.2	**17.7** ±19.5	**23.5** ±8.7
	*n*	5	15	14	14
	*t*-Test, *P*	*0.602*	*0.582*	*0.381*	***0.018***^c^
	M-W, P	*0.577*	*0.319*	***0.034***^c^	***0.039***^c^

SEM, Standard error of mean; *n*, number of repeats; *t*-Test, Student’s *t*-Test; M-W, Mann–Whitney *U*-Test; *P*, *P* value.

^a^*P* value, 0.001.

^b^*P* value, 0.01.

^c^*P* value, 0.05.

Since Pen-2 is required for the endocytic processing of PS-1 (53 kD) and activation of the γ-secretase complex [60], we investigated its protein levels following IL-18 addition. Pen-2 interacts with NTF but not with CTF of PS-1 [61]. However, Pen-2 (12 kD) was generally hardly detectable in most of the experiments (*n* = 9) at the 12 kD band, but at the highest concentration used (150 ng/mL) IL-18 appeared to increase its protein level at 72 h (Figure 2C). However, increased levels of Pen-2 antibody reactivity was found around the 80 kD band in IL-18 treated cells (Figure 2C,F). Aph1 and Nicastrin showed also some increase in 24-h IL-18 treated cells (Figure 2 F). Nevertheless, since the active γ-secretase complex includes the NTF and CTF (18.5 kD) of PS-1, Pen-2 (interacts with NTF), Aph1 (29 kD; interacts with CTF) and nicastrin (75 kD as unglycosylated), the high molecular weight bands shown in Figure 2B, C, and F are likely partially formed γ-secretase complexes. The approximate 100 kD band cluster (Figure 2B) was measured and its increase was 51% (*P* 0.016 (*t*-Test)) (Table 1) at 24 h after the IL-18 treatments.

Due to our earlier findings with GSK-3β [19], we also examined protein levels of Fe65. Fe65 (77 kD) interacts with AICD (Figure 1) and this complex regulates expression of GSK-3β [56], which in turn can regulate for instance the levels and function of PS-1 [57,58]. Protein levels of Fe65 were increased at 24 h in IL-18 treated cells, at IL-18 concentrations of 100 or 150 ng/mL (Figure 2D).

Expression of ADAM10, one of the α-secretases, was very low in our cells (Figure 2G) suggesting that it is not the main α-secretase in SH-SY5Y cells. Other α-secretases were not examined. However, it seemed that IL-18 treatment slightly decreased the 60 kD active form of ADAM10 levels when compared to actin (Figure 2G), but this result requires confirmation with other methods.

### IL-18 treatment increased levels of APP, altered its processing, and increased Aβ levels in SH-SY5Y cells

**Figure 3:**
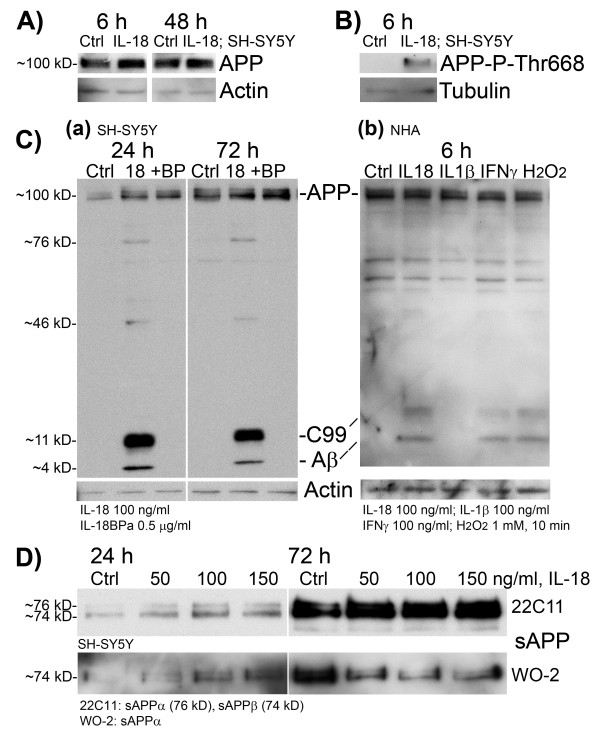
**Expression of APP products in differentiated SH-SY5Y neuroblastoma cells and in normal human astrocytes.** The differentiated SH-SY5Y-cells were treated with IL-18 (100 ng/mL) without or with IL-18 bp (0.5 μg/mL) for described times. IL-18 treatment increased (**A**) APP synthesis during 6-h and 48-h treatment and (**B**) Thr668 phosphorylation of APP during 6-h treatment in SH-SY5Y cells. IL-18 treatment also increased the protein levels of (**C**) C99 (approximately 11 kD) and Aβ (approximately 4 kD) (WO-2 antibody), the BACE-1 and γ-secretase cleavage products of APP in (a) differentiated SH-SY5Y cells but also in (b) normal human astrocytes. When examined from the same SH-SY5Y culture medium, (**D**) total sAPP was only slightly increased by the IL-18 treatment (22C11 antibody detects sAPPα (76 kD) and sAPPβ (74 kD)). When WO-2 antibody was used (detects only sAPPα) for the same samples, the protein level of sAPPα was less in IL-18 treated culture medium, compared to untreated control (Ctrl), showing indirectly the increase in sAPPβ production by IL-18 in SH-SY5Y medium.

IL-18 increased protein level of APP (75%, *P* 0.001) (Figure 3A, Table 1) as well as its phosphorylation at the Thr668 site (Figure 3B) in 6-h treated SH-SY5Y cells. Increase of APP was detectable also at the 48-h time-point (57%, *P* <0.000) (Table 1).

As detected by WO-2 antibody from the SH-SY5Y lysate, 100 ng/mL IL-18, for 24 or 72 h, increased the protein levels of APP cleavage products: Aβ (approximately 4 kD) and C99 (approximately 11 kD), as well as faintly expressed bands at approximately 76 kD (possibly sAPPα) and approximately 46 kD (possibly sAPP cleavage product (Figure 1)) as shown in Figure 3C(a). The effect of IL-18 was abolished with simultaneous treatment with 0.5 μg/mL of IL-18bpA (Figure 3C(a)), which is the predominant IL-18 bp form. Interestingly, IL-18 also increased levels of C99 and Aβ in NHA-cells treated for 6 h. IFN-γ (100 ng/mL) and 1 mM H_2_O_2_ (10 min insult) had a similar effect, whereas IL-1β had no impact (Figure 3C(b)). Nevertheless, in both cell types, the produced type of Aβ was mainly Aβ40 although some Aβ42 was also produced (ratio: approximately 3 Aβ42/100 Aβ40). The total amount of Aβ40 (cell lysate and medium) was approximately 260.7 pg in 10^5^ SH-SY5Y cells, treated with IL-18 for 72 h *vs.* approximately 229.9 pg in 10^5^ untreated cells (13.3%, SEM ±0.81; *n* = 3; *P* 0.004 (*t*-Test)). However, the Aβ40 estimate is very crude, due to the pronounced background in ELISA. Aβ42 levels were estimated to be approximately 7.14 pg/mL from the same samples, but no clear difference between IL-18 treatment and control were apparent partially due to the sensitivity limits of the ELISA kit.

In SH-SY5Y culture medium, the ratio of sAPPα and sAPPβ showed alteration during the IL-18 treatment when compared to the untreated control (Figure 3D). As indirectly shown (binding regions for the antibodies are shown in Figure 1), IL-18 treatment increased the proportion of sAPPβ from total sAPP, whereas in untreated control cell medium, the amount of sAPPα was greater, supporting the increased β-amyloidogenic processing of APP.

### IL-1β altered the expression levels of BACE-1, PS-1 and APP but differently to IL-18

**Figure 4:**
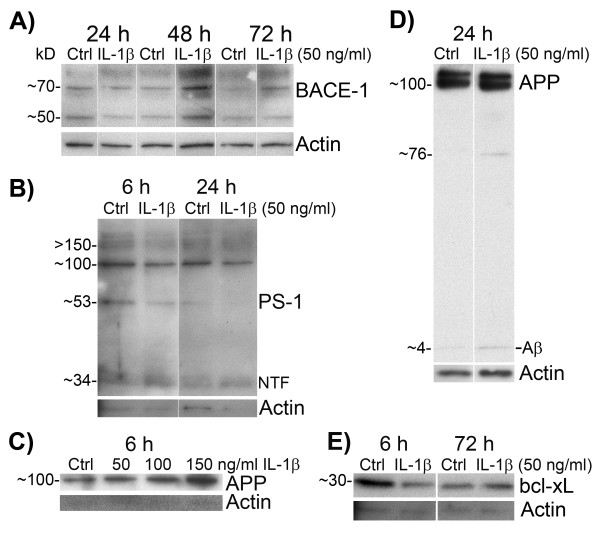
**IL-1β treated differentiated SH-SY5Y cells.** The cells were treated with IL-1β (50, 100, or 150 ng/ mL) for described times. (**A**) BACE-1 (70 kD) increased particularly in 48-hr IL-1β treatment (50 ng/mL) compared to the untreated control. Actin was used as loading control. (**B**) IL-1β increased protein level of NTF of PS-1 (34 kD), whereas protein synthesis of precursor PS-1 (53 kD) decreased in 6-h or 24-h IL-1β treated cells compared to untreated control. (**C**) IL-1β treatment increased protein levels of APP concentration dependently in 6-h treated cells. (**D**) IL-1β treatment increased slightly Aβ production. (**E**) Bcl-xL reduced in 6-h IL-1β treated cells but increased in the final 72-h time point. Ctrl, untreated control; NTF, N-terminal fragment of PS-1.

We treated differentiated SH-SY5Y cells also with IL-1β (10- to 150 ng/mL) as reference for IL-18. Since our main interest was IL-18, the number of repeats of these experiments was not enough to do proper statistics. However, changes were apparent mainly with 50 ng/mL or 100 ng/mL of IL-1β whereas 10 or 20 ng/mL was too low for our targets and 150 ng/mL seemed to be harmful to the cells. Nevertheless, the main increase of BACE-1 appeared later than with IL-18, at 48 h when 50 ng/mL of IL-1β was used (Figure 4A). The impacts of IL-1β on PS-1 were also less than that of IL-18 (Figure 4B). IL-1β treatment concentration dependently also increased APP levels during 6-h treatment (Figure 4C) and Aβ became apparent (Figure 4D) at 24 h. Similar kind of results were gained when IL-1β was used as 100 ng/mL. However, IL-1 is tightly regulated and we did not examine the expression levels for instance of IL-1 receptor (IL-1R), IL-1 receptor antagonist (IL-Ra), or IL-1 receptor accessory protein (IL-1RAcP). The low expression level of, for example, IL-1RI or IL-1RAcP may cause the poor response of SH-SY5Y to IL-1, whereas high expression of IL-1Ra or soluble IL-1RII, shed from the plasma membrane by BACE-1 [62], may inhibit IL-1 binding to its functional receptor and thus reducing the response of the cells.

### IL-18 treatment increased cellular level of bcl-xL but had only a minor impact on cellular caspase-3 and did not affect LDH activity in culture medium

Since IL-18 treatment increased Aβ levels in cell lysate, and binding of IL-18 to its receptor complex can lead to activation of JNK and MAPK p38 [63], in turn activating pro-apoptotic signaling pathways [64], we examined expression of pro-apoptotic caspase-3 and anti-apoptotic bcl-xL from the cell lysate as well as LDH activity from the culture medium.

**Figure 5:**
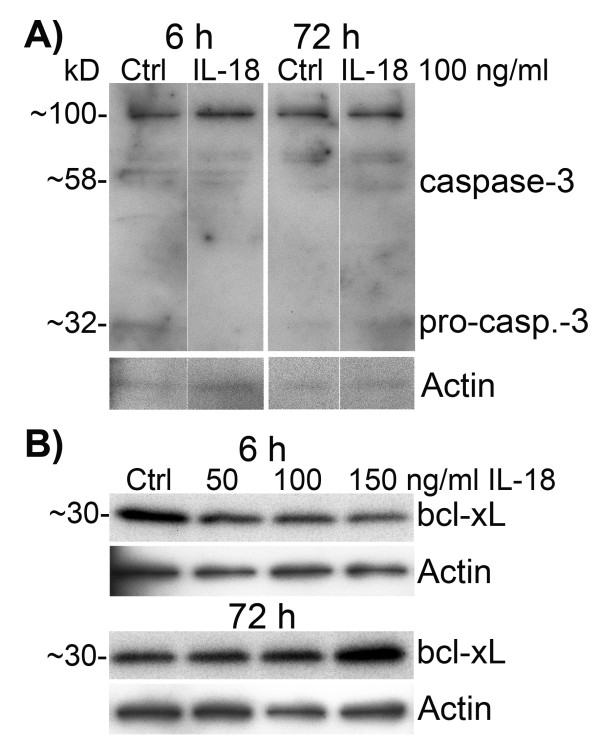
**Protein levels of indicators of apoptosis in differentiated SH-SY5Y cells.** The cells were treated with IL-18 (100 ng/mL) for described times. IL-18 (**A**) decreased pro-caspase-3 (approximately 32 kD) at 6 h, but increased its level in 72-h treated cells. The approximate 58 kD form, which is likely the active form composed of two 12 and 17 kD fragments of caspase-3, increased very slightly in 72-h treated cells. (**B**) Bcl-xL, which inhibits activation of caspases, was slightly reduced at 6-h IL-18 treated cells but induced in 72-h timepoint. Actin was used as a loading control.

**Figure 6:**
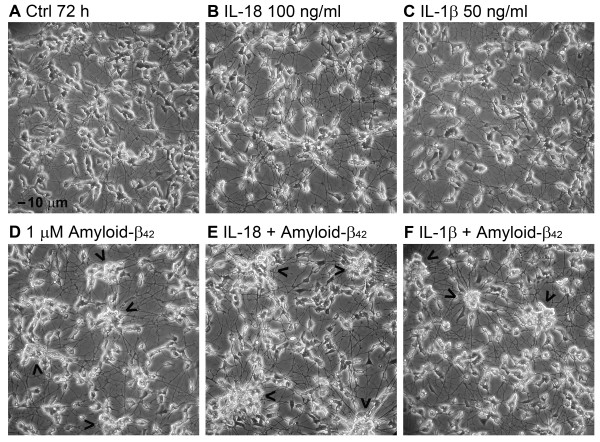
**The appearance of differentiated SH-SY5Y cells after IL-18 or IL-1β treatments with or without amyloid-β42 addition.** (**A**) Differentiated untreated control cells. (**B**) The cells were treated with IL-18 (100 ng/mL) or (**C**) with IL-1β (50 ng/mL) for 72 h. IL-1β exposed cells seemed to be more vacuolized than the control cells or IL-18 treated cells. The cells were treated (**D**) with 1 μM Aβ42 only, (**E**) with IL-18 (100 ng/mL), and 1 μM Aβ42 for 72 h, (**F**) with IL-1β (50 ng/mL) and 1 μM Aβ42 for 72 h. The arrowheads indicate the clustering of the cells. Magnification 200x, bar 10 μm as shown in (A).

Expression of pro-caspase-3 (32 kD) was reduced at 6 h in IL-18 treated cells, but increased at 72 h (Figure 5A). However, the protein level of caspase-3 (approximately 58 kD; approximate size of an active tetramer complex), showed only a minor increase at 72 h (Figure 5A). IL-18 also increased bcl-xL levels at 72 h (23%, *P* 0.039) (Table 1) in a concentration dependent manner (Figure 5B). The activity of LDH was not altered in culture medium even when IL-18 was used at 100 or 150 ng/mL for 72 h with or without 1 μM Aβ42 addition (data not shown). The survival of IL-18 or IL-1β treated with or without Aβ42 addition and untreated control cells showed no significant difference either, even after 72-h incubation (Figure 6) in all experiments (*n* = 6). However, Aβ42 treatment caused clustering of the cells, particularly when IL-18 was included (Figure 6E). Further studies with longer incubation time will be required to test for the differences, including vacuolization.

## Discussion

We found that IL-18 can increase production of APP and its Thr668 phosphorylation in neuron-like differentiated human SH-SY5Y cells. IL-18 also increased amyloidogenic processing to Aβ by inducing expression of BACE-1 and NTF of PS-1, part of the functional γ-secretase complex. IL-18 slightly increased protein levels of the anti-apoptotic bcl-xL and apoptotic caspase-3, but had no effect on LDH release.

The IL-18 increase in APP protein levels was an early response. APP, a member of the gene family including Amyloid Precursor-Like Proteins APLP1 and APLP2, exists as several distinct isoforms arising by alternative splicing of a single gene. The predominant neuronal APP isoform is APP695 [65]. APP has importance in cortical development, and mice that lack all three APP family members exhibit early postnatal lethality and neuroanatomical abnormalities [66]. APP may participate in regulation of the cell cycle during embryonic development [45], and seems required for correct neuronal migration [67]. APP forms are also associated with neurite outgrowth [68], with the cytoplasmic domain of APP interacting with the cytosolic adapter Fe65 in the regulation of neurite branching [69]. APP can also be rapidly transported down the axon to the synapses and, like Aβ, may have importance in the formation of synapses [70] and their functional regulation [71]. Thus, IL-18 may enhance neurite and synapse formation, which is in line with our earlier observations [19].

The IL-18 induced increase in APP Thr668 phosphorylation was an early response. APP has eight potential phosphorylation sites in its cytoplasmic domain [72], with its phosphorylation increasing in parallel with neuronal differentiation, especially at the time of neurite outgrowth [43]. Further, phosphorylated APP is distributed in neurites, particularly in growth cones [43]. Whereas GSK-3β can phosphorylate the cytoplasmic domain of the APP at its Thr743 [73], Cdk5, GSK-3α, and JNKs can mediate phosphorylation of N- and O-glycosylated APP at its Thr668 site [55,74]. Thr668 phosphorylation is linked to neurite extension and anterograde transport of vesicular cargo [75]. As such IL-18, via its modulation of APP, will impact on wide cellular processes, including synaptic plasticity. Further, Thr668 phosphorylation of APP facilitates its BACE-1 cleavage [54] aiding the initiation of the amyloidogenic pathway (Figure 1).

IL-18 also increased protein levels of BACE-1 (approximately 70 kD). BACE-1 is essential for cognitive, emotional, and synaptic functions [76]. Electrophysiologically, BACE-1-deficient neurons exhibit subtle alterations in the steady-state inactivation of voltage-gated sodium channels [77]. BACE-1 knockout mice in turn displayed significant neonatal lethality, suggesting an essential function. BACE-1 is highly expressed at the time when peripheral nerves become myelinated and is needed for neuregulin processing, with loss of BACE-1 inducing hypomyelination [78], suggestive of a role for IL-18 and BACE-1 in multiple sclerosis [79]. Further, experimental traumatic brain injury in rats also stimulates BACE-1 expression, production, and activity [80]. Thus, increased expression of BACE-1 by IL-18 suggests that BACE-1 and Aβ may be needed for synapse formation and sodium-voltage gate regulation during development as well as during regeneration. The role of IL-18 in the regulation of hypomyelination in neurodegenerative disorders requires further investigation.

The impact of IL-18 on PS-1 was not as pronounced as on APP and BACE-1. IL-18 increased precursor PS-1 (53 kD) moderately as well as Pen-2 (12 kD) very slightly, but later than the increase in APP and its Thr668 phosphorylation. The levels of endoproteolytic NTF of PS-1 significantly increased approximately at the time of the rise in APP and BACE-1. This suggests that the immediate interaction of Pen-2 with the PS-1 precursor leads to the endoproteolytic cleavage of PS-1 and to the formation of a functional γ-secretase complex. Despite this, in our experiments, the limiting factor for Aβ production seemed to be γ-secretase, since the level of C99 fragment (Figure 1) was generally higher than that of Aβ indicating the need of an additional stimulus for activation of γ-secretase. It has been shown that absence of PS-1 and PS-1/PS-2 strongly reduces Pen-2, which is further reduced when nicastrin level is low [81]. On the other hand, down-regulation of Pen-2 decreased PS levels and impaired nicastrin maturation, resulting in deficient γ-secretase complex formation. Thus the components of the γ-secretase complex are expressed in a coordinated manner [81]. Nevertheless, γ-secretase has an essential role in development and neurogenesis [82,83], but it is also a promiscuous aspartyl protease, which can cleave numerous type-I transmembrane proteins after their large ectodomain are shed [84,85]. The substrates of γ-secretase include proteins involved in neurite outgrowth and synapse formation as well as cell fate decisions and adhesion [84]. Presenilin has also other functions independent of γ-secretase, including roles in organelle transport and turnover, Akt-ERK signaling, cytoskeletal dynamics, and as a calcium leak channel in the endoplasmic reticulum [86].

The IL-18 induction of Aβ was abolished by the simultaneous treatment with IL-18 bp. Interestingly, IL-18 also increased Aβ production in normal human astrocytes similar to the effects of IFN-γ and H_2_O_2_, whereas IL-1β had only a very minor impact. The normal function of astrocytic Aβ is not understood, although glia, which are more abundant than neurons, may substantially contribute to the Aβ-plaque formation when the amyloidogenic cleavage system is activated in astrocytes. The Aβ-plaques are primarily made of Aβ peptides 1 to 40 and 1 to 42, both forms elevated in AD, but particularly Aβ42 is correlated with AD cognitive decline [87-89]. However, Aβ is also produced as part of normal metabolism and can be detected in the blood [90] and cerebrospinal fluid [91]. Physiological level of Aβ may regulate synaptic function and keep neuronal hyperactivity in check by negative feedback [92]. When accumulated inside the cells, Aβ is toxic [93,94] and can disrupt synaptic function [92]. Thus IL-18 in both neurons and astrocytes may contribute to enhancing Aβ effects, both positive and negative, particularly in the aged neurons.

Alterations in APP processing were also apparent in the culture medium. The ratio of sAPPβ to sAPPα increased following IL-18 treatment. sAPPα, reduced in familial and sporadic AD patients [95], has been implicated in neurite outgrowth [96], neuroprotection [97], neurotrophism and adult neurogenesis [98], axonal transport [99], synaptic function [92], and transcriptional regulation [100]. sAPPβ in turn can robustly drive neural differentiation of human embryonic stem cells [101]. Thus, IL-18, via sAPPβ to sAPPα ratio will modulate neural differentiation and survival functions.

The CTFs of APP, increased in AD patients’ brains, may be more neurotoxic than Aβ. These CTFs have been shown to impair calcium homeostasis as well as learning and memory through blocking LTP [102]. The cleavage of the C99 fragment through residues 48 to 50 by γ-secretase generates AICD59 (Figure 1), which can interact with several binders, including the phosphotyrosine interaction domain of Fe65 [72] to form a transcriptionally active complex. We showed that IL-18 increases protein level of Fe65 (approximately 77 kD). However, the phosphorylation of Thr668 in AICD is essential for its binding to Fe65 and to nuclear translocation, where it forms a ternary complex with transcription factor CP2/LSF/LBP1. This complex functions as a transcriptional activator for GSK-3β, APP, and BACE, as well as for transgelin, α2-actin, IGFBP3, and Aβ degrading neprilysin [103]. In our previous study [19] we found increased levels of GSK-3β after IL-18 treatments. Whether Fe65 mediated this remains to be determined. GSK-3β also regulates the CTF of PS-1 through its Ser397 phosphorylation [57], likely to decrease γ-secretase activity and altering the balance of C99 and Aβ.

The early phase of Aβ42-induced cytotoxicity in neuronal cells is associated with vacuole formation and enhancement of exocytosis [104]. A suggestion of increased vacuolization in IL-18 treated cells was apparent at the final time point. Since caspase-3 activation can be induced by Aβ leading to apoptosis [105], we also examined protein levels of caspase-3. Protein level of pro-caspase-3 (32 kD) altered time-dependently indicating its processing to the functional tetrameric complex. On the other hand, the tetrameric 58 kD form of caspase-3 increased only very modestly in IL-18 treated cells. Although caspase-3 is implicated in APP processing into amyloidogenic fragments with the accumulation of caspase-cleaved APP evident in the early phases of AD [52], the role of caspase-3 in APP processing when the amyloidogenic processing machinery is activated is unknown.

Bcl-xL is a negative regulator of caspase-3 activation in immature neurons during development [106]. Caspase-3 and bcl-xL also seem to have epistatic and independent functions in developmental programmed cell death [107]. Bcl-xL protects neurons from Aβ neurotoxicity, as shown in bcl-xL over-expressing SH-SY5Y cells [108]. In our studies, protein levels of bcl-xL were slightly increased at the final time-point by IL-18 treatment, suggesting a counteracting of caspase-3 apoptotic processes.

In conclusion, the present study shows that IL-18 increases APP expression and phosphorylation, which precede increased BACE-1 levels. The IL-18 induction of BACE-1, APP processing and Aβ is likely to be linked to stress associated adaptations in neurons and glia during the course of normal functioning and development. Further, in our cell culture system the limiting factor for Aβ production seemed to involve PS-1, the activation of which may require more enhanced expression of Pen-2 or other factors, influenced by other mediators than IL-18. However, in the course of wider changes in the aging brain, and particularly in AD brain, the effects of heightened or prolonged levels of IL-18 may contribute to the process of AD, including via increased Aβ.

## Abbreviations

Aβ, Amyloid-β; AD, Alzheimer’s disease; ADAM, A Disintegrin and Metalloproteinase domain-containing protein; AICD, Amyloid precursor protein Intracellular Cytoplasmic/C-terminal Domain; Aph, Anterior pharynx-defective; APLP, Amyloid precursor-like protein; APP, Amyloid precursor protein; ATRA, All trans retinoic acid; BACE-1, β-site amyloid precursor protein cleaving enzyme; bcl-xL, B-cell lymphoma-extra large; bp, Binding protein; Cdk5, Cyclin dependent kinase 5; CNS, Central nervous system; CTF, C-terminal fragment; ERK, Extracellular signal-regulated kinase; GSK-3, Glycogen synthase kinase-3; H_2_O_2_, Hydrogen peroxide; IGFBP, Insulin-like growth factor binding protein; IL, Interleukin; IL-18 bp, IL-18 binding protein; IL-18R, IL-18 receptor; IFN-γ, Interferon-γ; JNK, c-Jun N-terminal kinase; LDH, Lactate dehydrogenase; LPS, Lipopolysaccharide; LTP, Long-term Potentiation; NTF, N-terminal fragment; NF-κB, Nuclear factor κ-light-chain-enhancer of activated B cells; Pen-2, Presenilin enhancer 2; PS-1, Presenilin-1.

## Competing interests

The authors declare that they have no competing interests.

## Authors’ contributions

ES and JO carried out the cell culture, laboratory work, and analyses; JO, TP, AS, and ES contributed to the design of the study; TP and ES provided the research support. JO, ES, and GA wrote the manuscript. All authors read and approved the final version of the manuscript.
